# Diagnostic Accuracy of Cell Block and Immunohistochemistry in Effusion Cytology

**DOI:** 10.7759/cureus.34958

**Published:** 2023-02-14

**Authors:** Saima Batool, Safana Sadaf, Akhtar S Chughtai, Aafia Qasim, Asma Zafar, Anum Jamil

**Affiliations:** 1 Histopathology, Chughtai Institute of Pathology, Lahore, PAK

**Keywords:** hematoxylin and eosin, negative predictive value, positive predictive value, cell block, immunohistochemistry

## Abstract

Introduction

Although the cytology of effusion fluids is considered a routine laboratory test, it has recently emerged as an essential tool in determining the primary site of origin of carcinoma of unknown primary. The sensitivity for diagnosing malignancy has enhanced with the inclusion of cytospin, cell block (CB), and immunohistochemistry (IHC) to effusion fluid cytology due to the improvement in morphological preservation and good cellular yield. The purpose of this study was to assess the diagnostic yield, sensitivity, specificity, positive predictive value, and negative predictive value of IHC and CB in effusion cytology.

Methodology

An institution-based cross-sectional study was conducted over a period of six months on 150 cases of effusion fluids submitted for diagnostic purposes. After the preparation of cytospin, the residual amount of centrifuged deposit was mixed with CytoLyt solution, thrombin, and plasma, and CBs were prepared. IHC was applied to the CB. Calretinin was used for mesothelial cells, and BerEP4, TTF-1, ER, WT-1, and CD-X2 were used for the confirmation and origin of malignant cells.

Results

The mean age of the patients was 51.75 ± 16.63 years. The male-to-female ratio was 1:1.24. Out of 150 cases, 78 were pleural effusions, 68 were peritoneal effusions, and four were pericardial effusions. Out of 150 cases, based on cytological examination alone, 66 (44%) were classified as benign, 27 (18%) as malignant, and 57 (38%) were suspicious for malignancy. When cytology was combined with CB and IHC, the diagnostic yield was increased to benign 95 (63.33%), malignant 48 (32%), and suspicious for malignancy 7 (4.67%). The most common cause of malignant pleural effusion was breast carcinoma in females and lung carcinoma in males. The most common primary tumor in malignant peritoneal effusion was ovarian carcinoma in females and colonic adenocarcinoma in males. The sensitivity and specificity of combined cytology with cell block and IHC were 92.31% and 98.95%, respectively. This combination produced significantly better results (p-value = 0.001) for detecting malignancy and reduced suspicious cases from 38% to 4%.

Conclusion

CB, in combination with IHC, increases the diagnostic yield and aids in detecting malignancy at an unknown primary site in effusion fluids. Both of these techniques can thus enhance the sensitivity and specificity of the diagnosis of effusion cytology. Hence, CB and IHC have advanced utility over cytological smears in effusion fluid cytological diagnosis.

## Introduction

Cytological examination of effusion fluids is a routine laboratory test. However, it is now emerging as an essential tool in determining the primary site of origin of carcinoma of unknown primary. It not only plays a diagnostic role but also has prognostic significance and is an important tool in disease staging [1]. Because of increased cell yield and morphological preservation, the addition of cytospin and cell block (CB) techniques in effusion cytology have increased the sensitivity to diagnose malignancies. CB is used as an adjunct method for cytological diagnosis to distinguish between benign and malignant effusions. It is a quick and affordable technique that improves cell morphology preservation. Additional multiple levels of tissue, immunohistochemistry (IHC), and molecular testing can all be done on CB. The suggested method for fine needle aspiration cytology is CB [2]. The lab uses effusion fluid for cytological analysis, biochemistry, and the detection of cancerous cells. The leftover fluid, which is often thrown away after that, can be used to create a CB for a more accurate cancer diagnosis. Malignancy and the main site may be identified more precisely when the CB method and IHC are combined with a standard cytological smear [3]. Additionally, adjunctive methods such as IHC on CB can aid in differentiating between benign and malignant effusions [4]. Current effusion cytology practices state that there are numerous diagnostic difficulties. For instance, benign mesothelial cells may experience reactive alterations that mimic cancer. On the other hand, adenocarcinomas and malignant mesothelial cells might share many cytomorphological characteristics. It is crucial to make this distinction for both prognostic and diagnostic purposes. In order to distinguish between mesothelial and malignant cells, as well as between adenocarcinoma and mesothelioma cells, IHC may be employed as an auxiliary technique in challenging situations [5-7]. For this, a panel of antibodies may be utilized. This study aimed to determine the diagnostic yield, sensitivity, specificity, positive predictive value (PPV), and negative predictive value (NPV) of cytospin in conjunction with CB and IHC in effusion cytology.

## Materials and methods

A cross-sectional study was conducted at Chughtai Institute Of Pathology, Lahore, Pakistan, from January 2019 to July 2019 on 150 cases of effusion cytology whose samples were submitted for diagnostic purposes. An Institutional Review Board endorsed the study protocol at the Chughtai Institute of Pathology (reference letter number CIP/IRB/1003). The sampling technique was non-probability consecutive sampling. The inclusion criteria included fluids from patients aged 14 to 95 years, clinical details, and an adequate amount of fluid. Those fluids which were inadequate or less in quantity were excluded from the study. Out of these 150 cases, 78 were pleural, 68 peritoneal, and 4 were pericardial fluids. After the liquid was received in the department, a physical examination was conducted of the volume, color, consistency, and presence of the clot. After that, two cytospins were made for each sample and stained with Giemsa and pap stains. The remaining sediment was formalin-fixed and used to form CB using the plasma thrombin method. When preparing CB, the clot was taken into a tube with at least 2-3 ml of liquid. It was centrifuged for 2 to 3 minutes. The supernatant was discarded, and CytoLyt solution was added into the tube and left for 30 minutes. Normal citrate plasma was dispensed until the clot was absolutely immersed. The same quantity of thrombin was added to it and left for 2-3 minutes. A piece of wet filter paper was taken, and the block was put on it. Eosin was sprinkled, and it was placed on an embedding cassette. Then it was run on a tissue processor after fixation. The clot was fixed in a 10% neutral buffered formalin solution and automatically processed into a paraffin-embedded block. A histological slide was cut, and H&E staining was performed.

IHC was applied to all 150 cases of effusion cytology. It was performed on 3 um CBs using the complex streptavidin-biotin peroxidase technique. The staining was performed manually. Antigen retrieval was done using the heat-induced epitope retrieval method by the Dako PT Link instrument. Positive tissue controls for the histopathological section were used as controls. A panel of IHC markers included Calretinin, TTF-1, BerEP-4, ER, WT-1, and CDX-2 (Dako, Glostrup, Denmark). SPSS version 22 (IBM Corp., Armonk, NY, USA) was used in the data analysis. The numerical variables in the descriptive analyses were reported in the form of means and SDs, while the frequencies or categorical variables were reported in the form of percentages. Sensitivity, specificity, PPV, and NPV were calculated. P-value <0.05 was regarded as statistically significant.

## Results

One hundred fifty fluid samples from patients aged 14 to 95 years were included in this study. The maximum number of patients belonged to the age group 40-60 years, with a mean age of 51.75 ± 16.63 years. The male-to-female ratio was 1:1.24. There were 67 men (44.67%) compared to 83 women (55.33%). Among 150 cases, 78 (52%) were pleural effusions, 68 (45%) were peritoneal effusions, and 4 (3%) were pericardial effusions. Diagnosis of cytology and CB were classified as benign (negative for malignancy), malignant (clearly malignant cells present), and suspicious for malignancy (presence of cells with atypia not enough for a diagnosis of malignancy) according to cytomorphological and architectural features. Malignant pleural fluid cases were 27 (34.61%) after CB and IHC findings (Table 1).

**Table 1:** Diagnostic yield of cytology alone and with combined cell block and immunohistochemistry in pleural fluid (78 cases).

	Benign number (%)	Malignant number (%)	Suspicious number (%)
Cytology n =78	30 (38.46%)	15 (19.23%)	33 (42.31%)
Cytology, cell block, and immunohistochemistry n=78	47 (60.26%)	27 (34.61%)	4 (5.13%)

The most common cause of malignant pleural effusion was breast carcinoma in females (n=4, 14.81%) and lung carcinoma in males (n=11, 40.74%), followed by ovarian carcinoma (n=2, 7.40%), carcinoma of the stomach (n=2, 7.40%), malignant mesothelioma (n=1, 3.70%), and colonic adenocarcinoma (n=1, 3.70%). Other six (22.22%) cases remained undiagnosed for the primary site of origin, while only primaries screened for were excluded. Malignant peritoneal fluid cases were 21 (30.88%) after CB and IHC findings (Table 2).

**Table 2:** Diagnostic yield of cytology alone and with combined cell block and immunohistochemistry in peritoneal fluid (68 cases).

	Benign number (%)	Malignant number (%)	Suspicious number (%)
Cytology n=68	33 (48.53%)	12 (17.65%)	23 (33.82%)
Cytology, cell block, and immunohistochemistry n=68	44 (64.71%)	21 (30.88%)	3 (4.41%)

The most common cause of malignant peritoneal effusion was ovarian carcinoma in females (n=7, 33.33%) and colon carcinoma in males (n=4, 19.04%), followed by two cases of breast carcinoma (n=2, 9.52%), pancreaticobiliary carcinoma (n=2, 9.52%) and stomach carcinoma (n=1, 4.76%). Other five (23.81%) cases of malignant peritoneal fluid remained undiagnosed for the primary site of origin. All four (100%) cases of pericardial fluid proved to be benign after CB and IHC (Table 3).

**Table 3:** Diagnostic yield of cytology alone and with combined cell block and immunohistochemistry in pericardial fluid (four cases).

	Benign number (%)	Malignant number (%)	Suspicious number (%)
Cytology n=4	3 (75%)	0 (0%)	1 (25%)
Cytology, cell block, and immunohistochemistry n=4	4 (100%)	0 (0%)	0 (0%)

**Figure 1:**
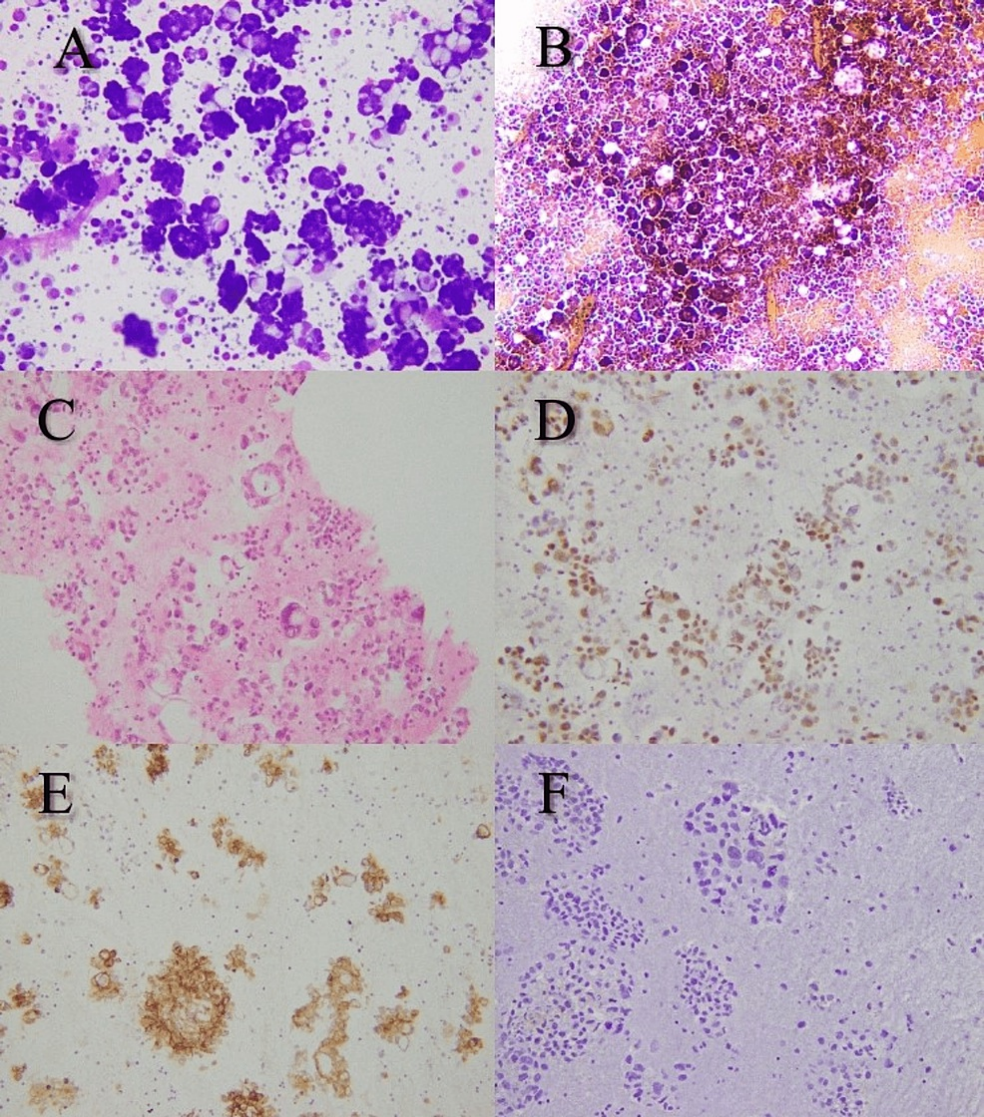
Metastatic lung adenocarcinoma in pleural fluid cytology. (A) Cytospin preparation shows tumor cells arranged in a three-dimensional configuration (Giemsa staining x400). (B) Cytospin preparation shows metastatic adenocarcinoma (Papanicoloau staining x200). (C) Cell block of the same patient with tumor cells clusters (H&E x400). (D) Immunohistochemistry for TTF-1 is positive (IHC staining x200). (E) BerEP4 IHC is positive (IHC staining x400). (F) Calretinin IHC is negative in tumor cells (IHC staining x400). IHC: Immunohistochemistry.

In breast carcinoma, ER IHC stain was positive, and WT1 was negative, favoring breast origin. In lung adenocarcinoma, TTF1 and BerEP4 positivity confirmed lung origin (Figure 1).

**Figure 2:**
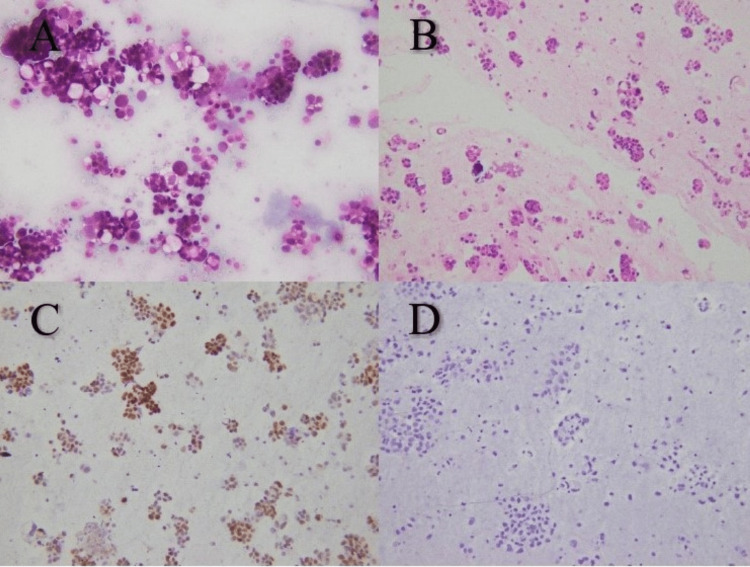
Metastatic serous carcinoma of ovary in peritoneal fluid cytology. (A) Cytospin preparation shows tumor cells arranged in smooth, three-dimensional cell ball and papillary-like configuration (Papanicoloau staining x400). (B) Cell block of the same patient with tumor cells in papillary configuration (H&E x200). (C) Immunohistochemistry for WT-1 is positive (IHC staining x200). (D) Immunohistochemistry for Calretinin is negative (IHC staining x200). IHC: Immunohistochemistry.

WT1 IHC positivity in peritoneal fluid pointed toward serous carcinoma of the ovary (Figure 2).

CDX2 positivity confirmed metastatic colon adenocarcinoma. Calretinin was performed to detect mesothelial cells in effusion cytology. BerEP4 was also positive in adenocarcinomas. In pleural fluid, out of 27 (100%) malignant cases, IHC proved to be useful in determining the site of origin in 21 (77.78%) cases. In contrast, in peritoneal fluids, 16 (76.19%) out of 21 (100%) malignant cases revealed the primary site of origin. Out of 150 cases on cytological examination, 66 (44%) were categorized as benign, 27 (18%) as malignant, and 57 (38%) as suspicious for malignancy on cytology alone. When cytology was combined with CB and IHC, the diagnostic yield was increased to benign 95 (63.33%), malignant 48 (32%), and suspicious 7 (4.67%) (Table 4).

**Table 4:** Diagnostic yield of cytology alone and with combined cell block and immunohistochemistry (total cases 150).

	Benign number (%)	Malignant number (%)	Suspicious number (%)
Cytology n=150	66 (44%)	27 (18%)	57 (38%)
Cytology, cell block, and immunohistochemistry n=150	95 (63.33%)	48 (32%)	7 (4.67%)

The sensitivity and specificity of combined CB and IHC were much more than those cytology alone, amounting to 92.31% and 98.95%, respectively. This combination produced significantly better results (p-value=0.001) for detecting malignancy and reduced the suspicious cases from 38% (n=57) to 4% (n=7) (Table 5).

**Table 5:** Diagnostic accuracy parameters of cytology with combined cell block and immunohistochemistry in effusion cytology.

Parameter	Percentage
Sensitivity	92.31%
Specificity	98.95%
Positive predictive value	97.96%
Negative predictive value	95.96%

## Discussion

The CB technique is one of the conventional methods used to evaluate effusion cytology. This technique necessitates 10% buffered formalin as a fixative, increasing the diagnostic yield through better preservation and cytomorphological details. The CB method enhances the sensitivity of diagnosis in effusion cytology. It can be used for a variety of levels of examination, including special and IHC stains. These stains are the most popular and easily accessible options, and they can be applied to formalin-fixed, paraffin-embedded sections [8]. Our study included a total of 150 effusion fluids, which comprised pleural, peritoneal, and pericardial fluids. Most patients were between the ages of 40 and 60 years, with a mean age of 51.75 ± 16.63 years. Males were n = 67 (44.67%), while females were n = 83 (55.33%). The male-to-female ratio was 1:1.24. In a study by Dermawan JKT and Policarpio-Nicolas ML [9], the patients' age range was 3-97 years (median, 65 years). Roughly equal proportions of patients were male (15411; 51%) and female (14674; 49%). Our study results are comparable with these findings. According to our study, breast cancer and lung adenocarcinoma were the most frequent causes of malignant pleural effusion in females and males, respectively. The most frequent diagnosis in peritoneal fluids was serous ovarian cancer in females and colon carcinoma in males. All four (100%) pericardial fluid cases proved to be benign. In a study by Dermawan JKT and Policarpio-Nicolas ML [9], the most common primary malignancy in pleural fluid in males was lung adenocarcinoma, followed by hematolymphoid and genitourinary cancer in males and breast cancer in females, followed by lung and ovary cancer in females. Hematolymphoid was the most frequent metastatic cancer in peritoneal fluid in men, followed by colorectal and gastric tumors, and ovarian carcinoma was the most prevalent cancer in women, followed by breast and gastrointestinal tumors. Our results are compatible with these findings. DiBonito L et al. [10] described the same results in malignant pleural fluids. Based solely on cytology, 66 (44%) of the 150 patients in our study that underwent cytological evaluation were categorized as benign, 27 (18%) as malignant, and 57 (38%) as suspicious. Diagnostic yield increased to benign 95 (63.33%), malignant 48 (32%), and suspicious 7 (4.67%) when cytology was paired with CB and IHC. In our study, fluid cytology with CB and IHC had a sensitivity and specificity of 92.31% and 98.95%, respectively. According to the study by Dekker A and Bupp PA, the number of suspected and positive fluids obtained with the combined CB-and-smear technique was double that of specimens examined in smears only [11]. This study's findings are consistent with ours.

The published findings show that both cytospin and CB provide greater architectural and cytological cellular features. These architectural alterations, such as acini, papillary structures, and cell ball formation, confirm malignancy and identify the main site of tumors [12, 13]. Our study showed papillary structures in serous carcinoma of the ovary and cell ball formation in various adenocarcinomas, for example, breast carcinoma. IHC is considered a simple, reliable, and commonly used technique for determining the primary site of malignancies and investigating the prognosis and progression of malignant tumors [14-16]. Several IHC stains, including TTF1, BerEP4, Calretinin, ER, CDX2, and WT1, were used in our study. Both malignant and benign mesothelial cells exhibit calretinin nuclear and cytoplasmic expression. TTF-1 nuclear expression is more suggestive of the lungs. Breast and ovarian carcinomas have ER nuclear positivity, whereas WT-1 nuclear expression in tumor cells favors ovarian carcinoma. Malignant mesothelioma can also test positive for it. The major location is the lower GI tract, according to CDX2 nuclear expression. Adenocarcinoma is favorably stained for BerEP4 in the membranes [17, 18]. In our study, IHC helped identify the site of origin in 21 (77.78%) of the 27 (100%) malignant cases in pleural fluids and 16 (76.19%) of the 21 (100%) cases in peritoneal fluids. After applying IHC to the 27 (100%) positive cases on the CB, Miachieo N et al. demonstrated in their study that the main site of malignancy was discovered in 19 (70.37%) cases; hence, IHC proved to be useful in identifying the precise site of origin of malignancy in most cases [18]. There were certain limitations to our research, such as the limited panel of IHC. Additionally, the outcomes may not accurately represent other regions because it was a single-center study.

## Conclusions

In conclusion, CB, in combination with IHC of effusion fluids, increases the diagnostic yield and detection of malignancy at an unknown primary site. CB provides better architectural and cytological details of fluid cytology. Both of these techniques can enhance the sensitivity and specificity of the diagnosis of effusion cytology. As a result, CB and IHC are more useful than cytological smears alone in diagnosing effusions.
